# A Photoactivatable Nanopatterned Substrate for Analyzing Collective Cell Migration with Precisely Tuned Cell-Extracellular Matrix Ligand Interactions

**DOI:** 10.1371/journal.pone.0091875

**Published:** 2014-03-14

**Authors:** Yoshihisa Shimizu, Heike Boehm, Kazuo Yamaguchi, Joachim P. Spatz, Jun Nakanishi

**Affiliations:** 1 WPI Research Center for Materials Nanoarchitectonics (MANA), National Institute for Materials Science (NIMS), Tsukuba, Ibaraki, Japan; 2 Department of New Materials and Biosystems, Max Planck Institute for Intelligent Systems, Stuttgart, Germany; 3 CSF Biomaterials and Cellular Biophysics, Max Planck Institute for Intelligent Systems, Stuttgart, Germany; 4 Department of Chemistry, Faculty of Science, Research Institute for Photofunctionalized Materials, Kanagawa University, Hiratsuka, Kanagawa, Japan; 5 Department of Biophysical Chemistry, University of Heidelberg, Institute for Physical Chemistry, Heidelberg, Germany; National Center for Scientific Research Demokritos, Greece

## Abstract

Collective cell migration is involved in many biological and pathological processes. Various factors have been shown to regulate the decision to migrate collectively or individually, but the impact of cell-extracellular matrix (ECM) interactions is still debated. Here, we developed a method for analyzing collective cell migration by precisely tuning the interactions between cells and ECM ligands. Gold nanoparticles are arrayed on a glass substrate with a defined nanometer spacing by block copolymer micellar nanolithography (BCML), and photocleavable poly(ethylene glycol) (Mw  =  12 kDa, PEG12K) and a cyclic RGD peptide, as an ECM ligand, are immobilized on this substrate. The remaining glass regions are passivated with PEG2K-silane to make cells interact with the surface via the nanoperiodically presented cyclic RGD ligands upon the photocleavage of PEG12K. On this nanostructured substrate, HeLa cells are first patterned in photo-illuminated regions, and cell migration is induced by a second photocleavage of the surrounding PEG12K. The HeLa cells gradually lose their cell-cell contacts and become disconnected on the nanopatterned substrate with 10-nm particles and 57-nm spacing, in contrast to their behavior on the homogenous substrate. Interestingly, the relationship between the observed migration collectivity and the cell-ECM ligand interactions is the opposite of that expected based on conventional soft matter models. It is likely that the reduced phosphorylation at tyrosine-861 of focal adhesion kinase (FAK) on the nanopatterned surface is responsible for this unique migration behavior. These results demonstrate the usefulness of the presented method in understanding the process of determining collective and non-collective migration features in defined micro- and nano-environments and resolving the crosstalk between cell-cell and cell-ECM adhesions.

## Introduction

Collective cell migration plays critical roles both in physiological and pathological processes [1], [2]. It is one of the most important properties for the formation and maintenance of organized structures in multicellular organisms. Generally, epithelial cells migrate collectively, whereas mesenchymal cells prefer to migrate as individuals. However, in some spatiotemporally limited situations in vivo, the cells aggressively ignore this rule. For example, changes in the collective characteristics of cells via epithelial-mesenchymal transition (EMT) or vice-versa (mesenchymal-epithelial transition, MET) is essential during embryonic development and morphogenesis [3]. Furthermore, cancer metastasis can be considered to be the loss of the collective features upon the escape from the original tissue and to the re-establishment of a new colony/focus in other tissues. Various soluble factors and the expression of several genes have been identified to regulate the decision to migrate collectively or individually [4], [5], but it has recently become clear that cellular niches, composed of extracellular matrices (ECMs) and the surrounding cells, also play important roles in the regulatory processes.

Early studies on cell-spreading behavior from spheroidal aggregates demonstrated that cell-ECM interactions and cell cohesiveness are inversely proportional to each other [6], [7] in an analogous fashion to simple wetting behavior of soft condensed matter [7], [8]. Based on the soft matter models, cells should migrate more collectively with decreasing cell-ECM interactions, and they should prefer non-collective migration on strongly adhesive surfaces. However, recent molecular biological studies provide more detailed information on the crosstalk between the cell-ECM and cell-cell adhesions [9] and imply the existence of complex regulatory mechanisms [10]. For example, it has been demonstrated that focal adhesion kinase (FAK), an essential mediator of signaling induced by integrin engagement with ECMs, plays conflicting roles in cell migration and metastasis; some papers report it is a positive regulator of cell migration and cancer metastasis, whereas others report the opposite function [10], [11]. Variations in the cadherin and integrin subtypes in the cells used in the studies or in the type of ECMs and the different degrees of ECM remodeling between the studies may be the source of these controversial outcomes [11]. Therefore, the contribution of cell-ECM interactions to the regulation of migration collectivity needs to be explored under more chemically and biochemically defined conditions.

In this regard, block copolymer micellar nanolithography (BCML) offers an ideal platform [12], [13]. In this method, gold nanoparticles are periodically arrayed on a glass substrate in a well-defined nanoscopic geometry and thereafter functionalized with cell-adhesive ECM ligands [13]. In contrast to the surfaces prepared by simple dilution of ligand molecules, this substrate allows for the precise non-stochastic control of ligand spacing and thereby enables the quantitative control of cell-ECM ligand interactions. In addition, matrix remodeling can be minimized by passivating the intervening glass regions with PEG and conjugating an ECM ligand via an ethylene glycol group. Therefore, the analysis of cell migration phenotypes on chemically defined cell-ECM ligand interactions becomes possible.

Scratch wound healing assay has been widely used to study cell migration in the laboratory to examine the contribution of soluble factors and gene transcription to cell migration. However, the difficulty in precisely controlling the wound geometry and the inevitable production of cellular debris prevents the precise control of the cellular micro- or nano-environment by this approach. Alternative methods based on mechanical barriers [14]–[16] or dynamic substrates [17]–[19] have been developed to overcome these drawbacks. These methods allow for the analysis of cell migration from and/or along controlled geometrical confinements with well-defined migration frontiers [14], [15], [17], [18], [20].The cells are initially confined within given micro-scale regions, either by surrounding the regions with a mechanical barrier_ENREF_10 or by micropatterning the cell adhesiveness of substrates. Subsequently, the migration of the cells is induced by removing the barrier or by activating the previously inert areas of the dynamic substrates using an external stimulus. The dependency of collective migration modes on geometrical constraints [21] and the role of intercellular physical forces in collective migration have been clearly demonstrated using these approaches [22], [23]. We also reported the impact of cell cluster geometry and incubation time on the frequency of leader cell appearance in collective migration using our original photoactivatable dynamic substrates based on photocleavable poly(ethylene glycol) (PEG) [24]. It should be noted that this high dependency of the collective characteristics on cellular microenvironments implies a need to analyze collective cell migration in defined cellular microenvironments and that the simple observation of the migration behavior of randomly attached cells could lead to complex experimental results.

In the present study, we combine photoswitchable ligands with a well-defined adhesive nanopatterned substrate in order to develop a method for analyzing collective cell migration with precisely tuned cell-substrate interactions. By the functionalization of quasi-hexagonally arranged gold nanoparticles with photocleavable PEG and a much smaller cRGD peptide, we expect the cRGD ligand to become accessible only when PEG is photocleaved in a similar fashion to our previous study [25]. The photochemical control of cell migration is also essential in exploring the impact of the biochemical nanopatterns on the substrate and the effect of cell cluster microscopic geometry on collective migration characteristics because cell migration can be remotely induced by photoirradiation, keeping the substrate nanostructures intact. Here, we focus on the collective migration of HeLa cells because these cells have been shown to both decrease and increase the collectivity of migration by knocking down or overexpressing proteins involved in integrin-mediated signaling [26], [27]. Therefore, we expected that this cell type would change their migration phenotype sensitively in response to changes in the cell-ECM ligand interactions.

## Materials and Methods

### Reagents and substrates

All reagents were purchased from Wako (Osaka, Japan) unless described otherwise. Photocleavable PEG12K was synthesized according to the procedure described in our previous work [25]. The cRGD ligand was purchased from Peptide Specialty Laboratories (Heidelberg, Germany). PEG2K-silane was synthesized according to the method reported previously [28].

**Figure 1:**
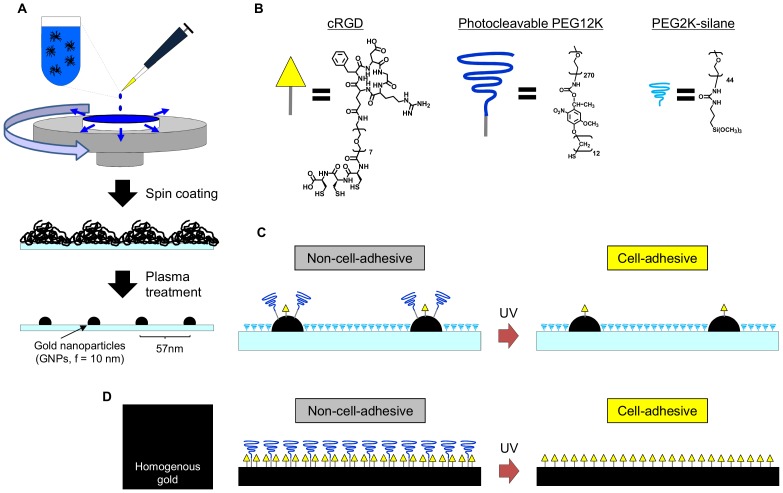
Schematic drawing of photoactivatable nanopatterned substrate. (A) The preparation of a nanopatterned substrate with periodically arrayed gold nanoparticles by block co-polymer nanolithography based on spin-coating. (B) Chemical structures of the cRGD ligand, photocleavable PEG12K and PEG2K-silane. The symbols used in C are also shown. (C) Photoactivation of the nanopatterned substrate. Before irradiation, the cRGD ligand is buried underneath the photocleavable PEG12K, and it is therefore not accessible to the cells. Near-UV irradiation releases the PEG12K, makes the cRGD ligand available in the nanoscopic geometry, and changes the surface from non-cell-adhesive to cell-adhesive. The intervening glass regions are passivated by PEG2K-silane. (D) Photoactivation of the homogenous substrate. An entirely gold-coated substrate was functionalized with a mixture of cRGD ligand and photocleavable PEG12K. The photoswitching strategy is the same as on the photoactivatable nanopatterned substrates, but this surface exposes cRGD ligand in a random geometry.

The chemical structures of the key components are shown in Figure 1B. Gold nanoparticles were arrayed on a glass coverslip based on block copolymer nanolithography [13]. Briefly, micellar solutions were prepared by dissolving 5 mg/ml polystyrene_1056_-poly[2-vinylpyridine]_495_ copolymers (Polymer Source, Canada) in *ο*-xylene (Merck) forming inverse micelles. After 24 h, HAuCl_4_·3H_2_O was added to the micellar solution with 0.2 equivalent per pyridine unit. The glass coverslips were cleaned with piranha solution (3∶1 H_2_SO_4_/H_2_O_2_) before spin-coating at 6000 rpm with the micellar solution followed by a treatment with W10 plasma (10% hydrogen, 90% argon) resulting in a quasi-hexagonal array of gold nanoparticles on the glass surface with an average particle size of 10-nm and 57-nm separations as determined by scanning electron microscopy (SEM). **Caution!** Piranha is a vigorous oxidant and should be used with extreme caution.

Before functionalization, the substrate was cleaned by a UV-ozone cleaner (UV253, Filgen, Nagoya, Japan) for 20–60 min. The glass regions of the nanopatterned substrate were passivated by immersing the substrate in a toluene solution containing 0.17 mM PEG2K-silane supplemented with a trace amount of triethylamine for 16 hr at 80°C. After the reaction, the substrate was rinsed and sonicated in toluene and methanol and then dried under nitrogen. An aliquot of an aqueous solution containing photocleavable PEG12K (15 μM) and cRGD (10 μM) was placed on a Teflon sheet, and the substrate was placed over the aliquot overnight under humidified conditions in the dark. After the reaction, the substrate was washed with pure water.

### Cell culture

HeLa cells were obtained from American type culture collection (ATCC). The cells were maintained in a state of continuous growth in MEM containing 10% FBS, 100 units/mL penicillin, and 100 μg/mL streptomycin at 37°C in a humidified atmosphere containing 5% CO_2_ and subcultured every 2 or 3 days using 0.25% trypsin-EDTA (Invitrogen, Carlsbad, CA). For the patterning experiments, the cells were harvested in phosphate-buffered saline (PBS, Takara Bio, Shiga, Japan) containing 5 mM EDTA in 37°C for 15 min.

### Scanning electron microscopy

The preparation of samples for scanning electron microscope observation was performed as described previously [29]. Briefly, the cells adhering to the photoirradiated nanopattern substrates were fixed with 4% glutaraldehyde in PBS for 15 min at room temperature. The specimens were washed with PBS and distilled water three times each and then dehydrated with serially increasing concentrations of ethanol (Wako) in water at 30, 40, 50, 60, 70, 80, 90, 95, 99, and 100%. The samples were washed with t-butyl alcohol three times and dipped in t-butyl alcohol. They were then frozen at 4 °C and freeze-dried using a vacuum pump. The samples mounted on an aluminum sample stub were coated with 2 nm of platinum using an E-1030 ion sputter (Hitachi, Tokyo, Japan), and observed with a scanning electron microscope (SU8000, Hitachi).

### Photopatterning

The photoactivatable nanopatterned substrate was placed in a glass-bottom dish (MatTek, Ashland, MA) in PBS and irradiated using an inverted fluorescence microscope (IX81-PAFM, Olympus, Tokyo, Japan) through an objective (UPlanSApo 10×, Olympus) and a band-pass filter (FF01-377/50, Semrock, Rochester, NY). The photoirradiation pattern was controlled by inserting a photomask made of a transparency at the position of the field diaphragm of the microscope [30], [31]. The photomask contained several circular regions with a ten-fold larger size in order to observe cell migration from multiple spots in one experiment. HeLa cells (1×10^6^ cells) were allowed to attach to the substrate in serum-free medium for 1 h, and the medium was changed to normal serum-containing medium to remove unattached floating cells from the culture chamber. The cells were maintained under these culture conditions for 8 h, and migration was induced by irradiating the regions surrounding the initial cell clusters.

### Time-lapse imaging

Time-lapse images of cell migration were obtained using a fluorescence microscope equipped with a charge-coupled device camera (Retiga-EXi, Q-Imaging), motorized stage (Molecular Devices, Downingtown, PA), an objective lens (UPLFLN10XPH, Olympus), and a heating chamber (INU-ONI-F1, Tokai Hit, Shizuoka, Japan) controlled by MetaMorph (Molecular Devices) software. The chamber was kept at 37°C in a humidified atmosphere containing 5% CO_2_. Several cell clusters were formed on the substrate, and phase-contrast images of the clusters were automatically taken every 5 min using MetaMorph.

### Cell migration tracking and quantification

Three or four representative cells, which were located at the periphery of the original circular clusters and which did not divide for 8 hours after the induction of cell migration were chosen. The contours of the cells based on the phase-contrast images were manually profiled every 30 minutes, and their centroids were determined using the MetaMorph software. The trajectories of the determined centroids during the 8-hour period were examined to evaluate the migration phenotypes as discussed below. The migration rate was calculated by dividing the summed distances between the consecutive centroid positions of the track by the time (8 hours), and the directional persistence was obtained as the net distance between the first and last positions of centroids divided by the summed distance. The means (± standard deviation) of these values were compared between the cells migrating on the homogenous and nanopatterned substrates, and the statistical significance of these means (P<0.05) was evaluated using two-tailed Student's t-tests.

### Immunofluorescence and image analysis

The following primary antibodies were used: mouse anti-N-cadherin, clone 32 (1∶100, BD Biosciences); mouse anti-vinculin (1∶800, Sigma); rabbit anti-FAK pY397 (1∶400, Life Technologies); rabbit anti-FAK pY861 (1∶100, Abcam); and mouse anti-FAK (H-1, 1∶100, Santa Cruz). Alexa Fluor 488 goat anti-rabbit IgG, and Alexa Fluor 546 goat anti-mouse IgG were used as secondary antibodies; both of these antibodies were purchased from Life Technologies. All antibodies were diluted with 1% bovine serum albumin (BSA) in PBS before use.

For the immunofluorescence observations, the cells were fixed for 20 min with 4% paraformaldehyde, washed with 5% glycine in PBS to quench free aldehydes, and permeabilized with 0.5% Triton X-100 in PBS. After blocking with 2% BSA in PBS, the cells were incubated for 2 hours with the primary antibodies and then incubated for 1 hour with the secondary antibodies. Finally, the substrates were washed with PBS and mounted on a glass coverslip with ProLong Gold antifade reagent (Life technologies). Actin filaments were stained with Alexa Fluor 568 phalloidin (1∶150; Life technologies). Epifluorescence images were obtained using a fluorescence microscope and analyzed with the MetaMorph software. The phosphorylation of Y397 and Y861 of FAK were determined by averaging the fluorescence intensity of 8–9 cells from each image. The statistical significance (P<0.01) was evaluated using two-tailed Student's t-tests.

## Results

### Surface design

**Figure 2:**
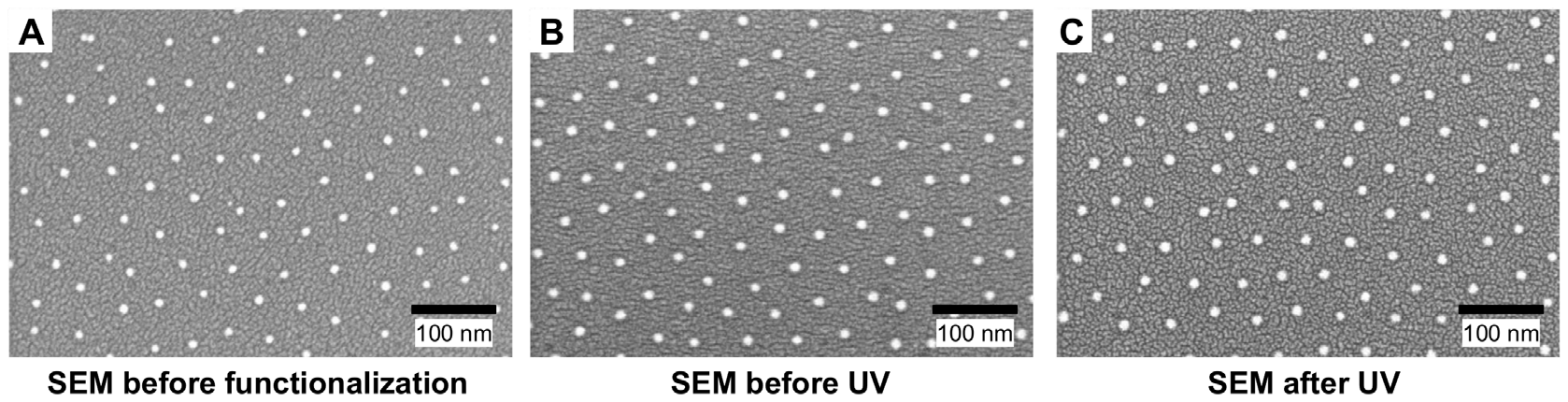
Functionalization and photoirradiation keep the nanoarrayed gold particles intact. (A–C) Scanning electron micrographs of (A) the as-prepared nanopatterned surface, (B) the chemically functionalized surface, and (C) the photo-irradiated surface. The scale bars represent 100 nm.

Nanopatterned substrates were prepared by block copolymer micellar nanolithography (Figure 1A). A chloroauric acid-loaded reverse micelle of diblock copolymer (poly(styrene-b-2-vinyl pyridine)) was prepared in toluene and spin-coated on a glass coverslip. The reverse micelle was arrayed on the surface in a quasi-hexagonal pattern with a given tens-of-nanometers spacing via the self-assembly process. The subsequent plasma treatment reduced the gold salt to nanoparticles and burnt away the coating polymer from the substrate. After the plasma treatment, the gold nanoparticles were embedded in the glass substrate, which was stable through the subsequent surface chemical functionalization and photoirradiation processes (Figure 2). In this study, we used a nanopatterned substrate with an average particle size of 10 nm and an inter-particle separation of 57 nm (center-to-center). The inter-particle distance was chosen based on the previous report [12], where MC3T3 cells spread well on the surface in a comparable level as that of a homogenous, non-nanopatterned surface. the selected particle size (10 nm) is sufficiently small, hence it is likely to accommodate single integrin heterodimers into each nanoparticle [32], enabling the “nano-digital” resolution of the cell-substrate interactions [29].

The arrayed gold nanoparticles and surrounding glass regions can therefore be functionalized with different chemical properties based on the gold-thiol (or gold-disulfide) and silane chemistries, respectively. Here, we functionalized the gold nanoparticles with a cRGD ligand (1.3 KDa) and photocleavable PEG12K and the functionalized surrounding glass regions with PEG2K-silane (Figure 1B). cRGD is a cyclic peptide containing the RGD sequence (named for the one-letter abbreviation of arginine-glycine-aspartate), which is an essential sequence in extracellular matrices (ECMs). The cRGD peptide mimics ECMs and interacts with integrins. In addition, due to the conformation constraint of the cyclic peptide, it has a high selectivity for specific integrin subtypes such as α_v_β_3_ [33]. The photocleavable PEG12K contains 2-nitrobenzyl ester, which is cleaved by near-UV irradiation. We have earlier demonstrated that an activity of a co-immobilized ligand, the Ni^2+^ complex of nitrilotriacetic acid (NTA), can be switched from the OFF state to the ON state by photoreleasing PEG12K. This is because its counterpart, a his-tagged protein, cannot access the Ni^2+^-NTA complex buried underneath the bulky PEG12K [25]. We expected that the same principle would be possible in controlling the activity of the cRGD ligand. The passivation of the glass regions with PEG2K-silane minimizes non-specific interactions on these regions, ensuring that the cells only interact with the surface via the ligands immobilized on the nanoparticles. Eventually, we are able to photocontrol cell adhesion on the surface, which is mediated by the nanoscopically presented cRGD ligands (Figure 1C).

### Photoswitchability of the nanopatterned substrate

**Figure 3:**
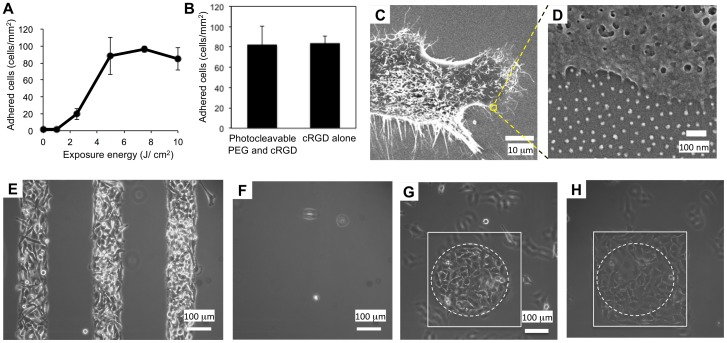
Photo-induced cell adhesion on the photoactivatable nanopatterned surface. (A) Dose-dependent increase of surface cell adhesiveness. The number of cells attached to a given area 3 hours after cell seeding is plotted against the irradiation energy. The error bars represent the standard deviations of the data from 3 different experiments. (B) Comparison of cell adhesiveness of the photoirradiated nanopatterned substrate to a similar surface where gold nanoparticles were functionalized with cRGD alone. The number of cells attached to a given area is plotted. The former surface was irradiated at 10 J before cell seeding. In either case, the glass regions were passivated with PEG2K-silane. The error bars represent the standard deviations of the data from 7 regions. (C, D) SEM images of a single HeLa cell migrating on the photoactivated nanopatterned surface. The image shown in D is a magnified image of the region indicated in C. (E, F) Photopatterning of HeLa cells in a stripe pattern on the nanopatterned surfaces, where gold nanoparticles were functionalized with (E) cRGD and photocleavable PEG12K or (F) photocleavable PEG12K alone. (G, H) Photoinduced cell migration. (G) HeLa cells were initially confined to a circular spot (indicated by the dotted line), and cell migration was induced by irradiating a square region (indicated by the solid line). A cellular image after 21 hours is shown in H. In all experiments, HeLa cells (4×10^5^) were allowed to attach to the surface in serum-free medium for 1 hour, and the unattached cells were removed when the medium was changed to normal serum-containing medium. For C-H, the surfaces were irradiated with near-UV light (λ = 365 nm, 10 J) in PBS before cell seeding.

We first evaluated the photoswitching ability of thus functionalized nanopattern in terms of surface cell adhesiveness. Figure 3A shows the number of attached cells at 3 hours after seeding versus the irradiated energy, the product of the irradiation power and time. The cells did not adhere to the original surface (0 J/cm^2^), but the number of attached cells increased with increasing irradiation energy up to a saturation level at 5.0 J/cm^2^. This profile is in good agreement with the PEG photoreleasing profile observed on an entirely gold-coated surface determined in our previous study [25], indicating a linear relationship between the conversion of the photochemical reaction and the change of cell adhesiveness. However, we used 10 J/cm^2^ throughout this study in order to ensure the completion of the reaction. This is because an insufficient photochemical reaction is unfavorable for exposing cRGD ligands on all of the arrayed nanoparticles. The cell adhesiveness of the irradiated surfaces showed no significant difference from that of a nanopatterned surface where the nanoparticles were functionalized with cRGD ligand alone (Figure 3B). In addition, nano-periodic cell-substrate contacts at cell peripheries were often observed by scanning electron microscopy (Figure 3C and 3D). These results indicate that the cRGD ligands become available at almost every nanoparticle after irradiating the surface with this dose.

We next tested our ability to control the cellular microenvironments on the substrate by geometrically confining cells within micrometer-scale regions. When the substrate was irradiated in a stripe pattern, the cells were selectively attached to the irradiated regions (Figure 3E). The cells were confined within the regions at least for one day (Figure 3D), which is sufficient for the purpose of the present study, where the cells were confine within the initial geometrical confinements for 9 hours. It is possible to confine the cells within the irradiated areas for up to 3 days based on our experiences in the surface PEGylation. The cRGD ligand is essential for the adhesion of HeLa cells to the irradiated regions because no cellular pattern was formed when the nanoparticled regions were functionalized with photocleavable PEG alone (Figure 3F). Therefore, all of the cellular phenotypes discussed in this study can be considered to be a result of the interactions between the cRGD peptide and the cells.

In order to demonstrate the application of this photoactivatable nanopatterned surface to cell migration assays, the HeLa cells were initially confined within a circular spot with a diameter of 270 μm (the dotted circle in Figure 3G), and their migration was induced by irradiating a larger square region (the square in Figure 3H). After the secondary irradiation, the cells started to migrate onto the newly formed cell-adhesive region, covering the whole irradiated region within 21 hours. As shown in our earlier studies [30], the substrate was irradiated for seconds by projection exposure through a photomask placed at the field diaphragm of a fluorescence microscope. This irradiation procedure allows us to maintain spatial control of the secondary irradiated regions and the initial cluster geometry down to a 5-μm resolution [20], and thereby enables the precise control of the cellular microenvironment in collective migration.

### Collective migration behavior of HeLa cells on differently adhesive substrates

**Figure 4:**
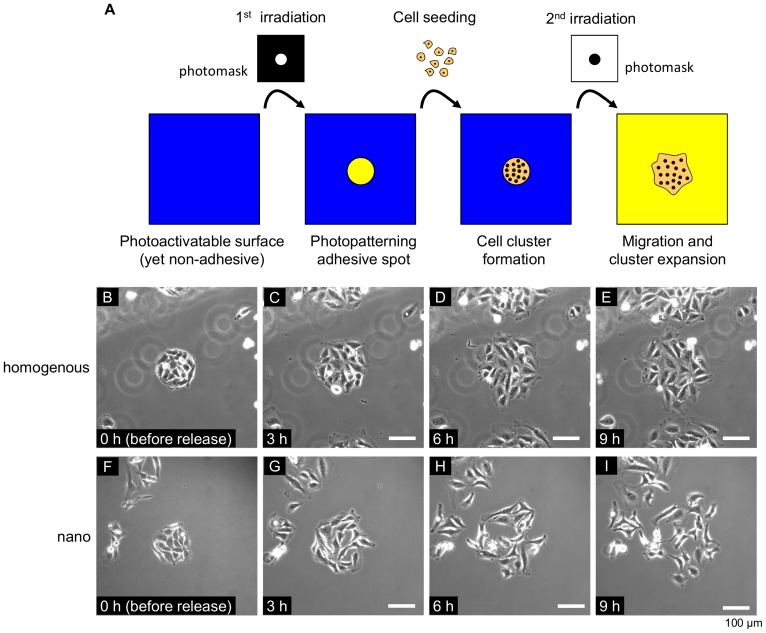
Cell migration behavior before and after release from geometrical confinement. (A) Schematic representations of the procedure for inducing cell migration on the photoactivatable homogenous and nanopatterned surfaces. The entire surface is initially non-cell-adhesive, and a 150-μm circular cell-adhesive spot is generated by the first irradiation. Cells are allowed to attach to the spot for 1 hour, and the unattached cells are removed by a medium exchange. The cells are further cultured for 8 hours to make a confluent circular cell cluster (B, F). The migration of the cells is induced by secondary irradiation through another photomask, which allows the selective irradiation of the surrounding regions (not over the patterned cells). The blue and yellow colors represent non-cell-adhesive and cell-adhesive surfaces, respectively. (B-I) Phase contrast images of the cells (B, F) before and (C, G) 3 hours, (D, H) 6 hours, and (E, I) 9 hours after the secondary irradiation on (B-E) the homogenous and (F-I) the nanopatterned gold substrates picked up from the supplemental movies (Movie S1, S2). The cells in the periphery of the images are nonspecifically attached cells or those have migrated from neighboring circles. The scale bar represents 100 μm.

As a control substrate, we prepared a homogenous, non-nanopatterned surface by functionalizing an entirely gold-coated substrate with the cRGD ligand and photocleavable PEG12K (Figure 1D). This substrate is also photopatternable (Figure 4B for example), but the cell-substrate interaction is mediated by the cRGD-integrin interaction in a random geometry. Furthermore, this homogenous surface can be assumed to be the ultimate case of the nanopatterned surface with no inter-particle separation. We then compared the cohesion properties of HeLa cells on these homogenously functionalized surfaces and the nanopatterned surfaces.

**Figure 5:**
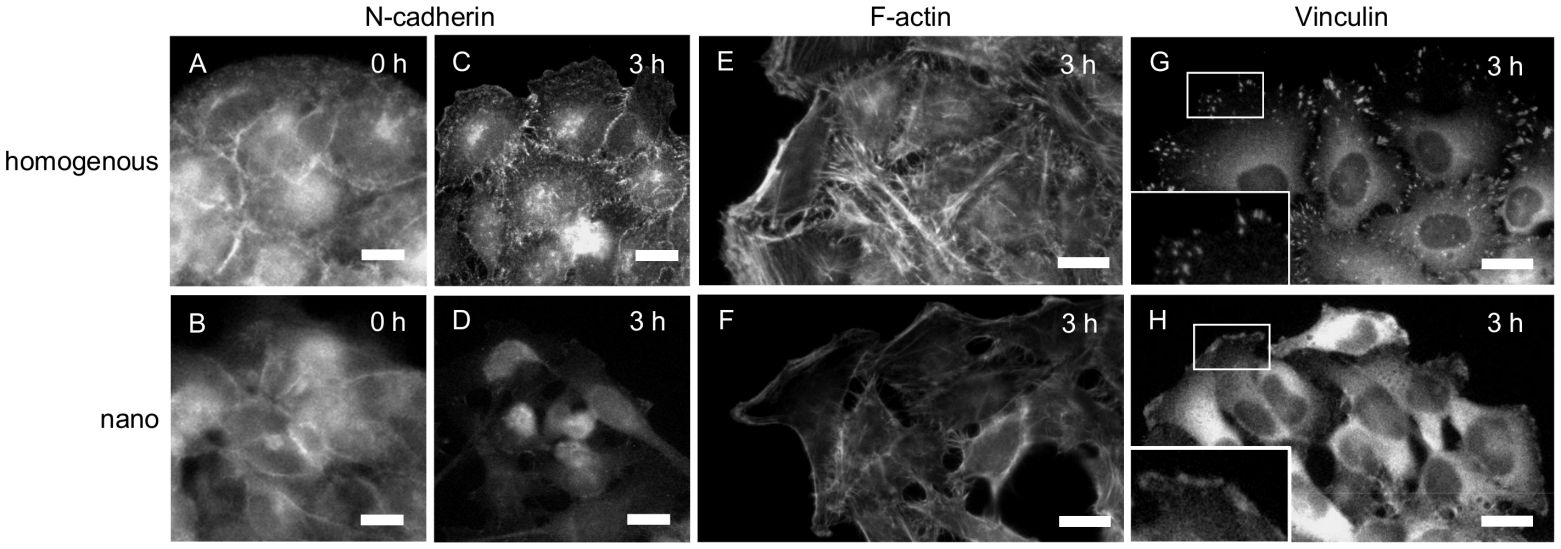
Immunofluorescence study of N-cadherin, cytoskeleton, and focal adhesion. HeLa cells were initially confined within a 150-μm circular spot on (A, C, E, G) the homogenous and (B, D, F, H) the nanopatterned surfaces for 9 hours and their migration was induced. The cells were fixed (A, B) before and (C-H) at 3 hours after the confinement release and stained for (A-D) N-cadherin, (E, F) actin, and (G, H) vinculin. The scale bars represent 20 μm.

Both the homogenous and nanopatterned substrates were irradiated in a circular spot with a diameter of 150 μm (Figure 4A). At nine hours after seeding, the HeLa cells became confluent within the initial circular cluster (Figure 4B and 4F).To ensure an almost identical microenvironment, we analyzed the results of cell migration from the spots whose initial cell number was between 20 and 25 cells/spot. N-cadherin, a cell adhesion protein, was localized along the cell-cell contact sites on both the homogenous and nanopatterned surface (Figure 5A, 5B). Cadherins are critical membrane proteins that form trans-homodimers between neighboring cells and hence play essential roles in maintaining cell-cell junctions during epithelial collective migration [34], [35]. In addition, we have demonstrated that a sufficient incubation time is required for the maturation of E-cadherin-mediated cell-cell contacts, when MDCK cells become capable of showing collective migration characteristics [24]. Taking these factors into consideration, we concluded that a 9-hour incubation is sufficient for cell-cell contacts to mature on either the homogenous substrate or the nanopatterned substrate.

**Figure 6:**
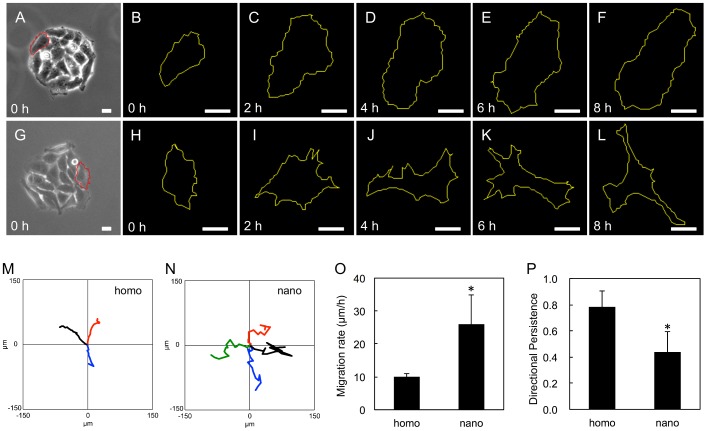
Tracking of migrating cells. (A, G) Circular clusters with a diameter of 150-μm formed on (A) the homogenous and (G) the nanopatterned substrates before the induction of migration. One representative cell which was originally located at the periphery of the cluster is chosen for each substrate and outlined with a red line. (B-F, H-L) The changes in the cellular shape shown in A and G at given time periods after migration induction. (M, N) The trajectories (black) of the cellular centroids of the cells indicated in A and G. The trajectories for 2 or 3 other cells in A and G are also shown, some of which are rotated to some extent so as not to overlap with other trajectories for convenience. (O, P) The averages of migration rate (O) and directional persistence (P) quantified from the centroid trajectories shown in M and N. The error bars represent the standard deviation for (M) 3 and (N) 4 cells. *Significant difference by a two-tailed Student's t-test (p<0.05). The scale bars represent 20 μm. The original images are taken from Movie S1 and S2.

However, when the cells were released from their confinements by irradiating the surrounding regions (Figure 4A), we observed dramatically different outcomes of cell migration on the two substrates (Figure 4B-4E vs. 4F-4I and Movie S1 vs. S2). On the homogenous surface, the cellular cluster expanded in a radial fashion toward the previously idle areas and maintaining the cell-cell contacts between the neighboring cells (Figure 4B-4E, Movie S1). The sustained cell-cell interactions during cell migration can also be clearly seen from the localization of N-cadherin at the sites of cell-cell contact (Figure 5C). Most of the cells formed a pancake-like shape and showed directed migration from the center of the cluster to outward (Figure 6A-6F, 6M). These cellular behaviors are similar to the sheet-like collective motions of epithelial cells during the wound healing process of scratched confluent monolayers [36]. On the other hand, on the nanopatterned substrate, the cells gradually became disconnected and migrated more individually (Figure 4F-4I, Movie S2). Even at 3 hours after the confinement release, the N-cadherin staining was mainly detected in the cytoplasm, and only trace signal or no signal was observed at the cell-cell contact regions on the nanostructured surfaces (Figure 5D), even though N-cadherin was localized at the cell-cell contact regions before the induction of migration (*vide supra*). When we focused on the individual cells each cell migrated back and forth, frequently changing its migration direction (Figure 6N and 6P). The cells gradually acquired elongated shape and extended more protrusions to random directions (Figure 6G-6L). The quantification of migration rate and directional persistence for the cells originally located at the periphery of the circular clusters are shown in Figure 6O and 6P. The data show that the average migration rate was significantly greater in the cells migrating on the nanopatterns than in the cells migrating on the homogenous substrates, whereas the average directional persistence was lower on the nanopatterned substrates. The final cellular phenotype of the cells migrating on the nanopatterned substrate was similar to that of HeLa cells with a depletion of a protein involved in integrin signaling, such as FAK, paxillin [26], and ZRP-1 [36], by RNA-interference. These results drove us to further examine the intracellular signaling induced by integrin.

### Structural and phosphorylation phenotypes of HeLa cells on the nanopatterned substrate

We first looked at the cytoskeletal structures and focal adhesion in cells migrating on the homogenous and nanopatterned substrates. The cells were fixed 3 hours after the induction of migration, and F-actin and vinculin were stained by using phalloidin and a corresponding antibody, respectively. As can be expected from the well-spread cellular shape (Figure 6B-6F), enhanced focal adhesion and stress fiber formation were observed in the cells migrating on the homogenously coated substrate (Figure 5E, 5G). In contrast, the actin cytoskeleton was mainly localized to the cell cortex regions, and few stress fiber was observed in the cells migrating on the nanopattern (Figure 5F). In addition, faint and blurry vinculin staining was observed in these cells (Figure 5H), indicating delayed or failure of focal adhesion maturation on the nanopattern. These observations are in clear contrast to the behavior of single fibroblast cells, which form stable and mature focal adhesions on similar nanopatterns [29]. The difference in the outcomes between previous and the current studies can presumably be attributed to different experimental conditions used in these experiments, such as, the cell types, cell densities, or different time ranges.

**Figure 7:**
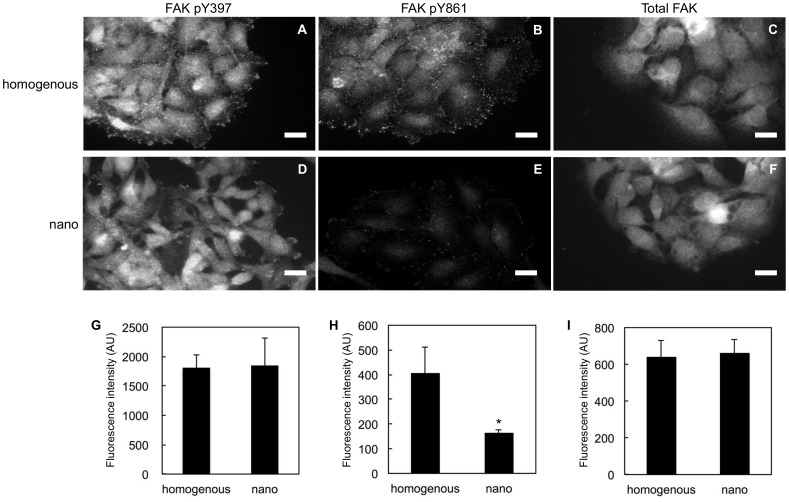
Phosphorylation of specific tyrosine residues on FAK in migrating cells. Immunofluorescence images for (A, D) pY397, (B, E) pY861, and (C, F) total FAK in cells migrating on (A-C) the homogenous and (D-F) nanopatterned surfaces 3 hours after the release from the initial confinement in the 150-μm circle. (G-I) The average fluorescence intensity of 8–9 representative cells shown in A-F. (G) pY397, (H) pY861, and (I) total FAK. The error bars represent the standard deviations. The scale bars represent 20 μm. *Significant difference by a two-tailed Student's t-test (p<0.01).

Poor focal adhesion maturation is often observed when the cells cannot receive sufficient integrin-mediated signaling. Focal adhesion kinase (FAK) is a non-receptor tyrosine kinase that plays critical roles in integrin-mediated signaling [37]. FAK undergoes phosphorylation on several specific tyrosine residues upon transmitting the signal from integrins. Earlier reports demonstrated that phosphorylation at Y397 and Y861, but not Y407 and Y925, of FAK is involved in the collective migration of HeLa cells [26]. Taking this into consideration, we focused on the phosphorylation at Y397 and Y861 of FAK in HeLa cells migrating on the homogenous and nanopatterned substrates using residue-specific phosphotyrosine (pY) antibodies. On either substrate, the fluorescence images showed punctate staining of pY397 throughout the cells with enhanced signal in the leading edge (Figure 7A, 7D). No statistically significant difference was observed in the level of pY397 estimated from the fluorescence intensity between the cells migrating on the homogenous and nanopatterned substrates (Figure 7G). In sharp contrast, the intensity of pY861 staining was globally weaker in the cells migrating on the nanopattern surface than in those migrating on the homogenous one, and the difference in these staining levels was statistically significant; however, the staining patterns were similar between the two surfaces (Figure 7B, 7E, and 7H). Immunofluorescence experiments based on an anti-FAK antibody representing the total FAK expression level showed no statistically significant variation in their staining intensities (Figure 7 C, F, and I), and therefore the observed difference in pY861 staining strongly indicates different degrees of phosphorylation on this tyrosine residue in cells migrating on different surfaces.

## Discussion

In this study, we developed a method to explore the impact of the cellular micro- and nano-environments on collective cell migration. The method is based on a photoactivatable nanopatterned substrate, which was developed by combining BCML and a photoswitchable ligand. It should be noted that a simple dilution of surface-immobilized cRGD ligands results in a large variation of the spacing between each ligand in the nanometer range, whereas BCML allows for extremely precise non-stochastic control of the spacing of ligands and thereby enables quantitative control of cell-substrate interactions. The particle size and the separation of the particles can be empirically tuned by selecting appropriate experimental conditions in the micelle formation and spin-coating. In addition, by replacing cRGD with appropriate peptide ligands or proteins [25], [38], we will be able to focus on the signaling induced by other integrin subtypes. Such analysis is of particular importance for the cells whose collective characteristics are regulated differently depending on the type of extracellular matrices [39]. Additionally, the photoswitch enables a micro-scale spatial resolution of cell clusters and the temporal resolution of the onset of cell migration, allowing for an analysis of collective cell migration in precisely controlled microenvironments. The high dependency of collective migration characteristics on the geometrical constraints observed in the earlier work [21], [24] strongly suggests that it is necessary to strictly control the cellular microenvironments. The presented system enables a well-controlled environment to focus on the influence of the cell-ligand interactions in collective migration without cross-talk caused by cell debris from scratching assays. Furthermore, it enables the systematic analysis of each of the soluble factors involved in wound closure in addition to the present research, which aims to elucidate the impact of the molecular interaction of cells with an ECM-derived peptide on the collectivity of migration using chemically defined nanopatterns.

Another important innovation of this work is the identification of a non-collective migration behavior of HeLa cells on the nanopatterned surface. It should be noted that, in the classical understanding, the cell adhesiveness of substrates and the collective migration characteristics of epithelial cells are believed to be inversely proportional to each other [6]. If we rely on this classical understanding and consider the nanopatterned surface simply as a weakly adhesive surface due to the lower surface cRGD density than the homogenous surface, the cells would migrate more collectively on the nanopatterned substrate. However, the result was the opposite of this expectation. At this time, we are not able to conclude whether the unique non-collective behavior observed in this study is specifically caused by the surface nanopatterns or is a unique intrinsic feature of HeLa cells. Given the observed gradual cellular morphology changes on the nanopattern (Figure 6H-6L), the cells may change their gene expression patterns and lose their collective characteristics as increasing the culture time on the nanopatterned substrate. Whatever the reason is, the immunofluorescence analysis based on the residue-specific phosphotyrosine antibodies of FAK clearly shows reduced phosphorylation of Y861 in the HeLa cells migrating on the nanopattern, whereas phosphorylation on Y397 was unchanged. Based on the work of Sabe and coworkers, the phoshporylation of Y861 is critical for the collective migration of HeLa cells [26]. pY861 attenuates the activity of the Rac small GTPase around the cell-cell contact regions to keep N-cadherin-mediated cell-cell junction. In addition, Y397 is known to be the first autophosphorylation site in response to the activation of FAK and Src tyrosine kinase recruited to the pY397 residue phosphorylates Y861 [37]. Therefore, the signaling between the phosphorylation of the Y397 and Y861 residues of FAK is hampered on the nanopatterned surface, and the reduced pY861 level results in the loss of the collective migration phenotype. Further molecular biological studies and spatiotemporally resolved studies using the photoactivatable nanopatterned substrates will hopefully elucidate the molecular mechanisms underlying this effect.

## Conclusions

Here, we report a photoactivatable nanopatterned substrate that is useful for the spatiotemporal resolution of the impact of the cellular micro- and nanoenvironment on collective cell migration. Interestingly, we observed the loss of collective migration characteristics in HeLa cells migrating on the nanopatterned surface. We can conclude that, at least in some limited situations, the cells lose (rather than gain) collectivity by decreasing interactions between cells and the ECM ligand, which opposites the conventional understanding. Immunofluorescence studies indicate that the defect in the tyrosine phosphorylation signaling between Y397 and Y861 is likely to be the source of the loss of collective characteristics in migration on the nanopattern. These results verify the usefulness of the presented material-based approach in exploring cellular nanoarchitectonics in collective cell migration, which will be complementary to conventional molecular biological methods.

## Supporting Information

Movie S1 **Time-course of a typical migration experiment on photoactivatable homogenous gold surface.** Movie shows HeLa cells seeded on the photopatterned homogenous surface with a 150-μm size circular spot and incubated for 9 h. Starting point is the time just after the cells were released from their confinement by irradiating the surrounding regions. Movie is shown in ×3000 speed. Duration 10 h; scale bar, 100 μm.
(MOV)

Movie S2 **Time-course of a typical migration experiment on photoactivatable nanopatterned substrate.** Movie shows HeLa cells seeded on the photopatterned nanopatterned surface with a 150-μm size circular spot and incubated for 9 h. Starting point is the time just after the cells were released from their confinement by irradiating the surrounding regions. Movie is shown in ×3000 speed. Duration 10 h; scale bar, 100 μm.
(MOV)
